# Draft Genome Sequence of *Vibrio parahaemolyticus* V110, Isolated from Shrimp in Hong Kong

**DOI:** 10.1128/genomeA.00300-13

**Published:** 2013-06-20

**Authors:** Ming Liu, Sheng Chen

**Affiliations:** Department of Applied Biology and Chemical Technology, The Hong Kong Polytechnic University, Hung Hom, Kowloon, Hong Kong; Food Safety and Technology Research Center, Hong Kong PolyU Shen Zhen Research Institute, Shenzhen, China

## Abstract

We report the whole-genome sequence of a *tdh*- and *trh*-negative *Vibrio parahaemolyticus* strain, V110, from shrimp. The major difference of V110 from clinical strains was its lack of the type III secretion system T3SS2, a key component of virulence. Further sequence comparison can shed light on the pathogenesis of *V. parahaemolyticus*.

## GENOME ANNOUNCEMENT

*Vibrio parahaemolyticus* is a Gram-negative marine bacterium that causes acute gastroenteritis related to the consumption of raw or undercooked seafood worldwide (1). In Hong Kong, *V. parahaemolyticus* is the leading cause of food-borne illnesses due to the high rate consumption of shrimp in that population. Epidemiological data showed that around 40% of food poisoning outbreaks in this city were associated with *V. parahaemolyticus*, especially in females aged 20 to 64 years old, who have the highest chance of contracting infections by this pathogen (2).

The study of the pathogenesis of *V. parahaemolyticus* has evoked considerable interest in recent years. Multivalent adhesion molecule 7 (MAM7), thermostable direct hemolysin (TDH), TDH-related hemolysin (TRH), and effectors secreted by type III secretion systems (T3SS) have been found with divergent virulence roles (3). For example, T3SS1 contributes to cytotoxicity, while T3SS2 is related to enterotoxicity. Recently, *in vivo* results have proven the critical role of T3SS2 on the colonization of the distal small intestine (4).

It was shown that T3SS1 is present in all *V. parahaemolyticus* strains, whereas T3SS2 seems to be frequently associated with only human clinical isolates. The environmental isolates have a much lower chance of carrying this transferable gene cluster, and those that harbor T3SS2 have different cytotoxicity and attachment rates to host cells (5). This indicates that virulence determinants are varied from strain to strain and that complicated genomic flexibility greatly affects their fitness to their host and their infection abilities. Therefore, it is necessary to decode the whole-genome sequence of more environmental isolates to better understand the relationship between pathogenicity and genome flexibility. Although there are nine available genome sequences of this species, only one of them is a nonclinical source (6–10). In the present study, the whole-genome sequence of *V. parahaemolyticus* strain V110, which was isolated from a shrimp purchased from a supermarket in Hong Kong in 2010, was decoded at the Beijing Genomics Institute (BGI)-Shenzhen, China.

Genomic DNA was extracted using a phenol-chloroform extraction method from strain V110 and was sequenced using Illumina HiSeq 2000. The sequencing library containing 500-bp inserts was constructed and sequenced with the pair-end strategy of 90-bp reads. A total of 549 Mb of filtered paired-end reads were generated. After assembly using SOAP*denovo* v1.05 (http://soap.genomics.org.cn/), 72 scaffolds and 405 contigs were produced. Gene prediction and genome comparison were determined using the RAST program (11). Genes encoding tRNA and rRNA were predicted through tRNAscan-SE and RNAmmer, respectively (12, 13). The total length of the draft genome is 5,583,571 bp, encoding 5,147 predicted open reading frames, 53 rRNA genes, and 66 tRNA genes with the average 45.2% G+C content. Unlike *V. parahaemolyticus* strain RIMD2210633, which has both T3SS1 and T3SS2, strain V110 contains only T3SS1. Intriguingly, strain V110 also possesses MAM7, an indicator of virulence potential. The roles of T3SS2, MAM7, and other genes unique to RIMD2210633 in the pathogenesis of *V. parahaemolyticus* will be investigated in the near future.

### Nucleotide sequence accession numbers.

This Whole-Genome Shotgun project has been deposited in DDBJ/EMBL/GenBank under the accession no. AQPJ00000000. The version described in this paper is the first version, accession no. AQPJ01000000.
